# Paraneoplastic and non-paraneoplastic autoimmunity to neurons in the central nervous system

**DOI:** 10.1007/s00415-012-6657-5

**Published:** 2012-09-15

**Authors:** Nico Melzer, Sven G. Meuth, Heinz Wiendl

**Affiliations:** 1 Department of Neurology, Inflammatory Disorders of the Nervous System and Neurooncology, University of Münster, Albert-Schweitzer-Campus 1, 48149 Münster, Germany; 2 Department of Physiology I, Neuropathophysiology, Robert-Koch-Strasse 27a, 48149 Münster, Germany

**Keywords:** Cancer, Autoimmunity, Neurons, Encephalitis, Antibodies, Cytotoxic T cells, Ion channels, Neurotransmitter receptors

## Abstract

Autoimmune central nervous system (CNS) inflammation occurs both in a paraneoplastic and non-paraneoplastic context. In a widening spectrum of clinical disorders, the underlying adaptive (auto) immune response targets neurons with a divergent role for cellular and humoral disease mechanisms: (1) in encephalitis associated with antibodies to intracellular neuronal antigens, neuronal antigen-specific CD8^+^ T cells seemingly account for irreversible progressive neuronal cell death and neurological decline with poor response to immunotherapy. However, a pathogenic effect of humoral immune mechanisms is also debated. (2) In encephalitis associated with antibodies to synaptic and extrasynaptic neuronal cell surface antigens, potentially reversible antibody-mediated disturbance of synaptic transmission and neuronal excitability occurs in the absence of excessive neuronal damage and accounts for a good response to immunotherapy. However, a pathogenic effect of cellular immune mechanisms is also debated. We provide an overview of entities, clinical hallmarks, imaging features, characteristic laboratory, electrophysiological, cerebrospinal fluid and neuropathological findings, cellular and molecular disease mechanisms as well as therapeutic options in these two broad categories of inflammatory CNS disorders.

## Introduction

The human central nervous system (CNS) can be targeted by aberrant cellular and humoral immune responses, which can either be triggered by systemic infections, and vaccinations (“postinfectious/-vaccinal autoimmune encephalitis”) and a variety of cancers (“paraneoplastic autoimmune encephalitis”) or occur without an (yet) identifiable cause (“non-paraneoplastic autoimmune encephalitis”) [25]. The scope of neurological disorders, in which such misguided adaptive immune responses are directed towards the oligodendrocyte and myelin-sheath is well described [24]. However, in a variety of immune-mediated CNS disorders, neurons seem to be targeted by adaptive cellular and humoral (auto) immune responses of both paraneoplastic and non-paraneoplastic origin [35]. Here, we summarize important clinical phenotypes together with their typical paraclinical measures, putative disease mechanisms, and therapeutic options in this emerging class of inflammatory CNS disorders.

## Prerequisites for neuron-directed autoimmunity in the CNS

Antigen-specific cellular and humoral immune responses directed towards CNS neurons are believed to develop as a multi-step process [73]. Soluble or cell-bound neuronal or neuronal-like antigens are engulfed and presented in the context of MHC II and co-stimulatory molecules to CD4^+^ T cells by professional antigen-presenting cell (APCs) within secondary lymphatic organs (e.g., cervical lymph nodes). This in turn permits CD4^+^ T cells via cytokine secretion and ligation of CD40 to license APCs to cross-present these antigens in the context of MHC I and co-stimulatory molecules to naive CD8^+^ T cells, which then become activated and may acquire cytotoxic effector functions (*cellular effectors*). A lack of such CD4^+^ T cell help usually results in anergy of CD8^+^ T cells. Depending on the local cytokine milieu mainly provided by CD4^+^ T cells, CD8^+^ T cells with different functional polarization may develop [86]. Tc1 cells are differentiated in the presence of IL-2 and IL-12, which induce the transcription factor T-bet. They produce IFN-γ and TNF-α and exert strong cytotoxicity. Tc2 cells are differentiated in the presence of IL-4, which induces the transcription factor GATA-3. They produce IL-4, IL-5, and IL-13 and exert a less robust cytotoxicity. Tc17 cells are differentiated in the presence of TGF-β and IL-6, which mainly induce the transcription factor RORγt. They produce IL-17 and have been reported to exert only weak cytotoxicity.

Naive B cells produce both IgM (and IgD) that are anchored in their plasma membrane and function as BCRs [48]. Naive B cells that encounter, ingest, and present their cognate so-called “thymus-dependent (TD) antigen” in the context of MHC II and co-stimulatory molecules to CD4^+^ T cells are in turn activated via cytokine secretion and ligation of CD40 and become antibody-secreting plasma cells (*humoral effectors*). Thereby, B cells can further diversify their Ig-genes by two DNA-modifying mechanisms [48]; somatic hypermutation and class switch recombination generate highly specific and adapted humoral responses. Somatic hypermutation introduces in a transcription-dependent manner non-templated point mutations in the variable (V) region of Ig genes, thereby enabling the selection of antibodies with increased affinity for the antigen. In contrast, class switch recombination modulates antibody effector function by replacing one constant (C) region with another, while retaining the binding specificity of the BCR. Depending on the cytokine milieu mainly provided by the CD4^+^ T cells, activated B cells undergo antibody class switching to produce IgG, IgA, or IgE antibodies [48]. Switch to IgG1 and IgG3 promoting complement activation and antibody-mediated cellular cytotoxicity by NK cells occurs in the presence of IFN-γ. In contrast, switch to IgG2, IgG4, and IgA promoting antigen-neutralizing effects occurs in the presence of IL-4 and IL-5. Although some pathogen-derived “thymus-independent (TI) antigens” may induce somatic hypermutation and class switch recombination in B cells independent from CD4^+^ T cell help, a lack of such help usually results in persistent secretion of complement-activating IgM (and IgD; [48]).

**Fig. 1:**
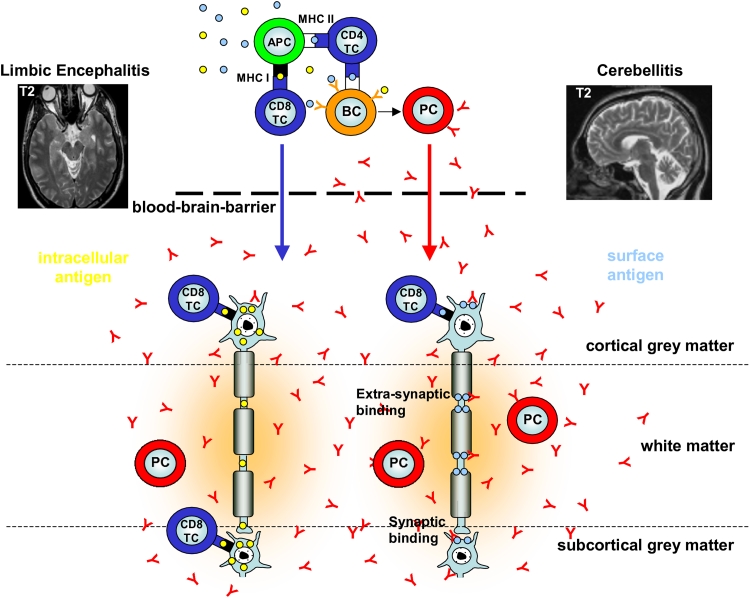
Putative immunopathogenesis of paraneoplastic and non-paraneoplastic auto-immunity to neurons in the CNS (modified and extended from Melzer et al. [70]). To set up an adaptive humoral and cellular immune response directed towards intracellular (*left, yellow circles*) or surface membrane (*right, bright blue circles*) neuronal antigen both professional antigen-presenting cells (APC, *green*) and naive antigen-specific B cells (BC, *orange*) need to encounter and engulf soluble or cell-bound neuronal or neuronal-like antigens within secondary lymphatic organs. Subsequently, APCs may process and present this antigen in the context of MHC II molecules to naive antigen-specific CD4^+^ T cells (CD4 TC, *blue*). MHC II-dependent recognition by these CD4^+^ T cells is then required for full activation of B cells to become antibody-secreting plasma cells (PC, *red*). In addition, CD4^+^ T cells may license APCs to present neuronal antigen peptides in the context of MHC I molecules also to naive CD8^+^ T cells (CD8 TC, *blue*) to acquire cytotoxic effector function. Antibody-secreting plasma cells and cytotoxic CD8^+^ T cells may invade the CNS to exert humoral and cellular neuron-directed autoimmunity

Following peripheral activation, both antibody-secreting plasma cells and cytotoxic CD8^+^ T cells (together with CD4^+^ T cells) may enter the CNS to attack neurons and cause functional and structural impairment [69, 97]. Moreover, under such inflammatory conditions, even antibodies produced in the periphery may permeate the blood–brain barrier (BBB) by various paracellular and transcellular mechanisms and thus contribute to neuron-directed immunity, whereas under physiological conditions, the BBB is usually impermeable for antibodies [23] (see Fig. 1).

In general, both effector arms of the adaptive immune response may be activated irrespective of the cellular localization of the neuronal antigen or its antigenic epitope (plasma membrane vs. interior cellular compartments). In terms of relevant effector mechanisms, plasma cell-derived antibodies usually recognize discontinuous conformational epitopes composed of segments of the respective neuronal plasma membrane protein antigen that are brought together in its three-dimensional structure and exposed on the neuronal plasma membrane. Antibodies may thus specifically impact the function and expression of theses antigens. Whether antibodies may also bind to and impact the function or expression of intracellular neuronal antigens, either by passive uptake into the neuron or by active binding to intracellular antigens that are transiently exposed to the plasma membrane is currently a matter of debate [30, 96]. Moreover, peptides derived from both intracellular and plasma membrane neuronal antigens might potentially be recognized by antibodies when exposed on the surface membrane in complex with MHC I molecules, although this is usually performed by CD8^+^ T cells.

Cytotoxic CD8^+^ T cells usually recognize continuous linear peptide epitopes consisting of 8–10 amino acids that are derived from intracellular neuronal proteins by extensive antigen processing and presented in the context of MHC I molecules on the cell surface membrane. Whether peptides derived from neuronal surface membrane antigens are also presented to cytotoxic CD8^+^ T cells in the context of MHC I molecules is unclear at present. In both cases, CD8^+^ T cells cannot directly impact the function or expression of their cognate antigens, but recognize their expression by the respective neuron. This enables them to contribute to neuronal dysfunction and cell death by the antigen-dependent release of effector molecules (perforin, granzymes) from cytotoxic granules. Indeed, we could show that two separate functional consequences result from a direct cell-to-cell contact between antigen-presenting neurons and antigen-specific CD8^+^ T cells. (1) An immediate impairment of electrical signaling in single neurons and neuronal networks occurs as a result of massive shunting of the membrane capacitance after insertion of channel-forming perforin (and probably activation of other transmembrane conductances), which is paralleled by an increase of intracellular Ca^2+^ levels. (2) Antigen-dependent neuronal apoptosis may occur independently of perforin and members of the granzyme B cluster, suggesting that extracellular effects can substitute for intracellular delivery of granzymes by perforin. Thus, electrical silencing is an immediate consequence of MHC I-restricted interaction of CD8^+^ T cells with neurons. Of course, these changes in neuronal excitability are not induced specifically in response to a certain antigen, but apply to all antigen-presenting neurons encountered by activated cytotoxic CD8^+^ T cells [69, 71].

## Paraneoplastic autoimmune encephalitis is probably mediated by cytotoxic CD8^+^ T cells specific for intracellular neuronal antigens

An ever-growing number of paraneoplastic CNS disorders are defined by the presence of IgG antibodies in the serum and CSF directed against intracellular neuronal antigens aberrantly expressed also by tumor cells (“onco-neuronal antibodies”) [67]. These tumors often contain neuronally differentiated tissue (germ cell tumors), express certain neuroendokrine peptides (SCLC, neuroblastoma), or occur in organs with a role in immune regulation (thymoma). However, due to the intracellular localization of the antigens, the humoral immune response is considered a non-pathogenic “epiphenomenon” solely indicating neuron-directed immunity and defining its antigen. In contrast, a variety of findings suggest a pathogenic role of cytotoxic CD8^+^ T cells for neuronal damage in these disorders: (1) neuronal damage often correlates with the number of CD8^+^ T cells, (2) CD8^+^ T cells are found in the CNS parenchyma in close spatial proximity to neuronal target cells, (3) CD8^+^ T cells show an activated phenotype with substantial expression of the effector molecules (perforin and granzymes) in cytotoxic granules with a polar orientation towards the target cell membrane, (4) CD8^+^ T cells stain positive for CD107 indicating recent exocytosis of cytotoxic granules (i.e., degranulation), (5) neuronal target cells exhibit substantial cell surface expression of MHC I molecules allowing for cognate antigen-recognition by CD8^+^ T cells, (6) CD8^+^ T cells exhibit a restricted T cell receptor (TCR) repertoire (i.e., oligoclonal expansions) suggesting that they have expanded from a few precursors locally responding to a distinct neuronal antigen [6, 69]. These criteria, however, have not yet been demonstrated entirely for all entities.

In clinical terms, inflammatory CNS disorders associated with IgG antibodies against intracellular neuronal antigens are characterized by a multifocal presentation of CNS-related symptoms involving the neocortex, the limbic system, basal ganglia, brainstem, cerebellum, and spinal cord as well as PNS-related symptoms involving radices, plexus, and peripheral nerves in a variable extent (Table 1). The clinical presentation partially reflects the pattern of expression of the respective neuronal antigen: ANNA-1 targets nuclear ELAVL (“Hu”) proteins expressed in central and peripheral neurons, and the corresponding clinical syndrome typically includes CNS and PNS manifestations [58]. In contrast, ANNA-2 targets nuclear NOVA-1 and -2 (“Ri”) proteins expressed in central, but not peripheral neurons and the clinical syndrome is usually restricted to the CNS [75] (Table 1). MRI findings include T2/FLAIR hyperintense, occasionally contrast-enhancing lesions in the cortex, medial temporal lobes, basal ganglia, brainstem, cerebellum, and spinal cord. Inflammatory changes are usually found in CSF studies including lymphocytic pleocytosis, mildly elevated protein together with intrathecal IgG synthesis and oligoclonal bands, but normal glucose and lactate levels [19]. The disease entities usually exhibit a chronic progressive clinical course and poor response to immunotherapy, especially to antibody-depleting therapies. Even successful removal of the tumor considered to drive the pathogenic immune response is usually not associated with disease amelioration [19].

**Table 1:** Encephalitis associated with antibodies against intracellular neuronal antigens

Entity	Patients	Triggers	Clinical hallmarks	Imaging	Electrophysiology	Laboratory	
ANNA-1 (Hu) encephalitis	Age 30–80 years (median 60 years), gender male 75 %, ANPR >500	Tumors: lung (SCLC in adults), neuroendocrine tissue (neuroblastoma in children), rarely thymus (thymoma)	Neocortical and limbic encephalitis, brainstem encephalitis, cerebellitis, myelitis, cranial neuropathy, radiculopathy, plexopathy, peripheral (sensory, motor, sensorimotor, autonomic) neuropathy	MRI: T2/FLAIR hyperintense signal, occasionally Gd-enhancement and atrophy in cortex, medial temporal lobes, brainstem, cerebellum or spinal cord FDG-PET: focal hypermetabolism at early disease-stages, focal hypometabolism at late disease-stages	EEG: 1. focal or widespread interictal and ictal epileptiform activity, 2. focal or generalized slowing Nerve conduction studies: predominantly axonal sensory, motor or sensorimotor neuropathy, plexopathy, radiculopathy	Anti-neuronal nuclear IgG1/3 antibody type 1 (ANNA-1; anti-Hu antibody) in serum and CSF targeting nuclear ELAVL (“Hu”) proteins expressed in central and peripheral neurons and tumor cells and implicated in neuronal post-transcriptional RNA regulation (“onco-neuronal” antibodies)	
ANNA-2 (Ri) encephalitis	Age 50–80 years (median 65 years), gender female 80 %, ANPR 100	Tumors: lung (SCLC), breast	Neocortical and limbic encephalitis, brainstem encephalitis, cerebellitis, myelitis	MRI: T2/FLAIR hyperintense signal, occasionally Gd-enhancement and atrophy in cortex, medial temporal lobes, brainstem, cerebellum or spinal cord FDG-PET: focal hypermetabolism at early disease-stages, focal hypometabolism at late disease-stages	EEG: 1. focal or widespread interictal and ictal epileptiform activity, 2. focal or generalized slowing	Anti-neuronal nuclear IgG1/3 antibody type 2 (ANNA-2; anti-Ri antibody) in serum and CSF targeting nuclear NOVA-1 and –2 (“Ri”) proteins expressed in central but not peripheral neurons and tumor cells and implicated in regulation of alternative splicing of neuronal RNA encoding synaptic proteins (N-, P/Q-type Ca^2+^ channels; “onco-neuronal” antibodies)	
ANNA-3 encephalitis	Age 10–85 years (median 60 years), gender female 50 %, ANPR 10	Tumors: lung (SCLC, adenocarcinoma), esophagus (adenocarcinoma)	Limbic encephalitis, brainstem encephalitis, cerebellitis, myelitis, peripheral (sensory, sensorimotor) neuropathy	MRI: T2/FLAIR hyperintense signal, occasionally Gd-enhancement and atrophy in medial temporal lobes, brainstem, cerebellum or spinal cord FDG-PET: focal hypermetabolism at early disease-stages, focal hypometabolism at late disease-stages	EEG: 1. focal or widespread interictal and ictal epileptiform activity, 2. focal or generalized slowing Nerve conduction studies: predominantly axonal sensory, motor or sensorimotor neuropathy	Anti-neuronal nuclear IgG1/3 antibody type 3 (ANNA-3) in serum and CSF targeting a nuclear 170 kDa protein of unknown molecular identity expressed in central (Purkinje neurons) and peripheral neurons and tumor cells (“onco-neuronal” antibodies)	
AGNA (SOX-1) encephalitis	–,	Tumors: lung (SCLC)	Limbic encephalitis, brainstem encephalitis, cerebellitis, peripheral neuropathy Lambert-Eaton myasthenic syndrome (LEMS)	MRI: T2/FLAIR hyperintense signal, occasionally Gd-enhancement and atrophy in medial temporal lobes, brainstem or cerebellum FDG-PET: focal hypermetabolism at early disease-stages, focal hypometabolism at late disease-stages	EEG: 1. focal or widespread interictal and ictal epileptiform activity, 2. focal or generalized slowing Nerve conduction studies: predominantly axonal sensory, motor or sensorimotor neuropathy EMG: decrement of compound muscle action potential on 2–5/s repetitive nerve stimulation, increment of compound muscle action potential on 30–50/s repetitive nerve stimulation or “post-tetanic” stimulation	Anti-glial/neuronal nuclear IgG1/3 antibody (AGNA) in serum and CSF targeting nuclear SOX1 protein expressed predominantly in developing and adult cerebellar Bergmann glia cells, central and peripheral neurons and tumor cells and implicated in transcription regulation during neuronal development (“onco-neuronal” antibodies)	
	ANPR 100						
Anti-Ma1/Ma2 encephalitis	Age 40–70 years (median 55 years), gender male 75 %, ANPR 75	Tumors: women with non-germ cell tumors of ovary, breast, colon, lung (combined anti-Ma1/Ma2-encephalitis), men with germ cell tumors of testis (pure anti-Ma2-encephalitis)	Anti-Ma1/Ma2 encephalitis: limbic encephalitis, diencephalitis, brainstem encephalitis and cerebellitis Anti-Ma2 encephalitis: limbic encephalitis, diencephalitis, brainstem encephalitis without cerebellitis	MRI: T2/FLAIR hyperintense signal, occasionally Gd-enhancement and atrophy in medial temporal lobes, diencephalon, brainstem or cerebellum FDG-PET: focal hypermetabolism at early disease-stages, focal hypometabolism at late disease-stages	EEG: 1. focal or widespread interictal and ictal epileptiform activity, 2. focal or generalized slowing	Anti-Ma1 (PNMA1)- and/or Anti-Ma2 (PNMA2) IgG1/3 antibody in serum and CSF targeting nucleolar/subnuclear Ma1 (PNMA1) and Ma2 (PNMA2) proteins expressed in central neurons and in tumor cells and implicated in transcription regulation (“onco-neuronal” antibodies)	
Anti-CV2 (CRMP-3) encephalitis	Age 50–75 years (median 60 years), gender female 60 %, ANPR 30	Tumors: lung (SCLC), thymus (thymoma), kidney (carcinoma), thyroid gland (carcinoma)	Uveitis, retinitis, optic neuritis, limbic encephalitis, cerebellitis, myelitis peripheral (sensory, motor, sensorimotor) neuropathy neuromyelitis optica-like clinical phenotype (optic neuritis + myelitis)	MRI: T2/FLAIR hyperintense signal, occasionally Gd-enhancement and atrophy in optic nerve, medial temporal lobes, cerebellum or spinal cord FDG-PET: focal hypermetabolism at early disease-stages, focal hypometabolism at late disease-stages	EEG: 1. focal or widespread interictal and ictal epileptiform activity, 2. focal or generalized slowing Nerve conduction studies: axonal and demyelinating sensory, motor or sensorimotor neuropathy	Anti-collapsin response-mediated protein 3 IgG1/3 antibody (CRMP-3-IgG) in serum and CSF targeting cytoplasmic collapsin response-mediated protein 3 expressed in a subpopulation of oligodendrocytes and central neurons, Schwann cells and peripheral neurons and tumor cells and implicated in axon guidance, synaptic organization and other cellular responses (“onco-neuronal” antibodies)	
Anti-CRMP-5 encephalitis	Age 50–75 years (median 60 years), gender female 60 %, ANPR 150	Tumors: lung (SCLC), thymus (thymoma), kidney (carcinoma), thyroid gland (carcinoma)	Optic neuritis with and without retinitis and other cranial neuropathies, neocortical and limbic encephalitis, “basal ganglionitis”, cerebellitis, myelitis, radiculopathy, plexopathy, peripheral (sensory, motor, sensorimotor, autonomic) neuropathy, myasthenia gravis (MG), Lambert-Eaton myasthenic syndrome (LEMS), neuromyotonia neuromyelitis optica-like clinical phenotype (optic neuritis + myelitis)	MRI: T2/FLAIR hyperintense signal, occasionally Gd-enhancement and atrophy in optic and other cranial nerves, neocortex, medial temporal lobes, basal ganglia, cerebellum or spinal cord FDG-PET: focal hypermetabolism at early disease-stages, focal hypometabolism at late disease-stages	EEG: 1. focal or widespread interictal and ictal epileptiform activity, 2. focal or generalized slowing Nerve conduction studies: axonal and demyelinating sensory, motor or sensorimotor neuropathy	Anti-collapsin response-mediated protein 5 IgG1/3 antibody (CRMP-5-IgG) in serum and CSF targeting cytoplasmic collapsin response-mediated protein 5 expressed in central and peripheral neurons including synapses and tumor cells and implicated in axon guidance, synaptic organization and other cellular responses (“onco-neuronal” antibodies)	
Anti-PCA-1 (Yo) encephalitis	Age 60–70 years (median 65 years), gender female 90 %, ANPR 150	Tumors: breast, ovary, fallopian tube, endometrium	Cerebellitis, brainstem encephalitis, myelitis, peripheral (sensory, motor, sensorimotor, autonomic) neuropathy	MRI: T2/FLAIR hyperintense signal, occasionally Gd-enhancement and atrophy in cerebellum, brainstem and spinal cord FDG-PET: focal hypermetabolism at early disease-stages, focal hypometabolism at late disease-stages	EEG: Generalized slowing or normal Nerve conduction studies: predominantly axonal sensory, motor or sensorimotor neuropathy	Purkinje cell cytoplasmic IgG1/3 autoantibody type 1 (PCA-1; anti-Yo antibody) in serum and CSF targeting cytoplasmic CDR2 and CDR62 (“Yo”) proteins expressed in central and peripheral neurons especially cerebellar Purkinje neurons and tumors cells and implicated in downregulation of transcription via inhibition of c-Myc (“onco-neuronal” antibodies)	
Anti-PCA-2 encephalitis	Age 45–85 years (median 60 years), gender female 70 %, ANPR 10	Tumors: lung (SCLC)	Limbic encephalitis, brainstem encephalitis, cerebellitis, peripheral neuropathy	MRI: T2/FLAIR hyperintense signal, occasionally Gd-enhancement and atrophy in medial temporal lobes, brainstem or cerebellum FDG-PET: focal hypermetabolism at early disease-stages, focal hypometabolism at late disease-stages	EEG: 1. focal or widespread interictal and ictal epileptiform activity, 2. focal or generalized slowing Nerve conduction studies: predominantly axonal sensory, motor or sensorimotor neuropathy	Purkinje cell cytoplasmic IgG1/3 autoantibody type 2 (PCA-2) in serum and CSF targeting a cytoplasmic 280 kDa protein of unknown molecular identity expressed in central and peripheral neurons especially cerebellar Purkinje neurons and tumors cells (“onco-neuronal” antibodies)	
Anti-PCA-Tr encephalitis	Age 15–70 years (median 60 years), gender male 75 %, ANPR 120	Tumors: Hodgkin lymphoma, non-Hodgkin lymphoma, occasionally solid tumors	(Limbic encephalitis), cerebellitis	MRI: T2/FLAIR hyperintense signal, occasionally Gd-enhancement and atrophy in cerebellum FDG-PET: focal hypermetabolism at early disease-stages, focal hypometabolism at late disease-stages	EEG: generalized slowing or normal	Purkinje cell cytoplasmic IgG1/3 autoantibody type Tr (PCA-Tr) in serum and CSF targeting Delta/Notch-like epidermal growth factor-related receptor (DNER) expressed in central neurons especially in cerebellar Purkinje neurons and occasionally in tumor cells (Reed-Sternberg cells) and implicated in neuron–glia interactions through notch signaling (“onco-neuronal” antibodies)	
Anti-amphiphysin encephalitis	Age 50–80 years (median 65 years), gender female 60 %, ANPR 100	Tumors: lung (SCLC, non-SCLC), breast, melanoma	Limbic encephalitis, cerebellitis, myelitis, stiff-person syndrome, radiculopathy, plexopathy, peripheral (sensory, motor, sensorimotor) neuropathy	MRI: usually normal, occasionally T2/FLAIR hyperintense signal, Gd-enhancement and atrophy in medial temporal lobes, cerebellum and spinal cord	EEG: Usually normal, occasionally focal or generalized epileptiform activity and slowing Nerve conduction studies: predominantly axonal sensory, motor or sensorimotor neuropathy EMG: excessive startle response, continuous involuntary motor activity in agonistic and antagonistic muscles	Anti-Amphiphysin IgG antibody in serum and CSF targeting cytoplasmic amphiphysin expressed in central and peripheral neurons (presynaptic terminals) and in tumor cells and implicated in retrieving vesicle membranes from the axon terminal’s plasma membrane after depolarization-induced exocytosis of neurotransmitter (“onco-neuronal” antibodies)	
Anti-GAD 65 encephalitis	Age 15–80 years (median 60 years), gender female 80 %, ANPR 200	Tumors (occasionally): lung (SCLC, non-SCLC), thymus (thymoma), colon, pancreas, breast, thyroid, and renal cell carcinoma	Limbic encephalitis, epilepsy, basal ganglionitis, brainstem encephalitis, cerebellitis, myelitis, stiff-person syndrome, Progressive Encephalomyelitis with Rigidity and Myoclonus (PERM) stiffness/rigidity, excessive startle, brainstem dysfunction (anti-GAD 65 IgG antibody titer usually >2000 U/ml in RIA) Autoimmune diabetes mellitus (anti-GAD 65 IgG antibody titer usually <20 U/ml in RIA)	MRI: usually normal, occasionally T2/FLAIR hyperintense signal, Gd-enhancement and atrophy in medial temporal lobes, brainstem, cerebellum and spinal cord	EEG: Usually normal, occasionally focal or generalized epileptiform activity and slowing EMG: excessive startle response, continuous involuntary motor activity in agonistic and antagonistic muscles	Anti-GAD 65 IgG antibody in serum (>20,00 U/ml) and CSF, usually with intrathecal anti-GAD 65 IgG synthesis targeting the cytoplasmic 65 kDa isoform of glutamic acid decarboxylase expressed in central GABAergic neurons (presynaptic terminals), pancreatic islet cells and occasionally tumor cells and implicated in converting excitatory neurotransmitter glutamate to inhibitory neurotransmitter GABA (occasionally “onco-neuronal” antibodies)	
Anti-GABA_A_-receptor-associated protein encephalitis	–,	–	Neocortical encephalitis, epilepsy, cerebellitis, stiff-person syndrome	–	EMG: continuous involuntary motor activity in agonistic and antagonistic muscles	Anti-GABARAP IgG antibody in serum and CSF targeting cytoplasmic and membrane GABARAP expressed in central neurons (postsynaptic density of GABAergic synapses) and implicated in clustering and anchoring GABA_A_-receptors in the postsynaptic membrane by facilitating binding to the cytoskeleton	
	ANPR 20						
Anti-gephyrin encephalitis	–,	Tumor: undifferentiated mediastinal carcinoma	Stiff-person syndrome	MRI: normal	EMG: continuous involuntary motor activity in agonistic and antagonistic muscles	Anti-gephyrin IgG antibody in serum and CSF targeting cytoplasmic gephyrin protein expressed in central neurons (postsynaptic density of GABAergic and glycinergic synapses) and implicated in clustering and anchoring GABA_A_- and glycine receptors in the postsynaptic membrane by facilitating binding to the cytoskeleton	
	ANPR : 1						

Entity	Patients	CSF	Neuropathology	Putative disease mechanisms	Therapy	Disease course and prognosis	References
ANNA-1 (Hu) encephalitis	Age 30–80 years (median 60 years), gender male 75 %, ANPR >500	Lymphocytic pleocytosis (median 3/μl, range 1–8/μl), normal glucose and lactate, mildly elevated protein (median 78 mg/dL, range 49–135 mg/dL), intrathecal IgG synthesis and OCB	Inflammatory infiltrates (perivascular CD4^+^ T cells and B cells, parenchymal CD8^+^ T cells), gliosis, microglia activation, neuronal loss and neuronophagia	Neuronal cell death mediated by neuronal-antigen specific cytotoxic CD8^+^ T cells	Immunotherapy: 1. GCS together with steroid-sparing agents, IVIG, PE/IA, 2. rituximab, cyclophosphamide; Tumor-therapy: (operation, radiation, chemotherapy)	Chronic disease course: usually poor response to immunotherapy; some improvement or stabilization may be achieved by prompt tumor-therapy	[1, 33, 37, 49, 58, 67, 79, 83, 85, 87]
ANNA-2 (Ri) encephalitis	Age 50–80 years (median 65 years), gender female 80 %, ANPR 100	Lymphocytic pleocytosis (median 5/μl, range 1–15/μl), normal glucose and lactate, mildly elevated protein (median 55 mg/dL, range 39–415 mg/dL), intrathecal IgG synthesis and OCB	Inflammatory infiltrates (perivascular CD4^+^ T cells and B cells, parenchymal CD8^+^ T cells and macrophages), gliosis, microglia activation, neuronal loss and neuronophagia	Neuronal cell death mediated by neuronal-antigen specific cytotoxic CD8^+^ T cells	Immunotherapy: 1. GCS together with steroid-sparing agents, IVIG, PE/IA, 2. rituximab, cyclophosphamide; Tumor-therapy: (operation, radiation, chemotherapy)	Chronic disease course: usually poor response to immunotherapy and tumor-therapy	[37, 59, 67, 75, 79, 83, 85]
ANNA-3 encephalitis	Age 10–85 years (median 60 years), gender female 50 %, ANPR 10	Lymphocytic pleocytosis, normal glucose and lactate, mildly elevated protein, intrathecal IgG synthesis and OCB	–	Presumably neuronal cell death mediated by neuronal-antigen specific cytotoxic CD8^+^ T cells	Immunotherapy: 1. GCS together with steroid-sparing agents, IVIG, PE/IA, 2. rituximab, cyclophosphamide; Tumor-therapy: (operation, radiation, chemotherapy)	Chronic disease course: usually poor response to immunotherapy and tumor-therapy	[12, 37, 67, 83, 85]
AGNA (SOX-1) encephalitis	–,	Lymphocytic pleocytosis, normal glucose and lactate, mildly elevated protein, intrathecal IgG synthesis and OCB	–	Presumably neuronal cell death mediated by neuronal-antigen specific cytotoxic CD8^+^ T cells	Immunotherapy: 1. GCS together with steroid-sparing agents, IVIG, PE/IA, 2. rituximab, cyclophosphamide; Tumor-therapy: (operation, radiation, chemotherapy)	Chronic disease course: usually poor response to immunotherapy and tumor-therapy	[36, 37, 67, 79, 83, 85, 92]
	ANPR 100						
Anti-Ma1/Ma2 encephalitis	Age 40–70 years (median 55 years), gender male 75 %, ANPR 75	Lymphocytic pleocytosis (median 3/μl, range 1–20/μl), normal glucose and lactate, mildly elevated protein (median 53 mg/dL, range 39–70 mg/dL), intrathecal IgG synthesis and OCB	Inflammatory infiltrates (perivascular CD4^+^ T cells and B cells, parenchymal CD8^+^ T cells and macrophages), gliosis, microglia activation, neuronal loss and neuronophagia	Neuronal cell death mediated by neuronal-antigen specific cytotoxic CD8^+^ T cells	Immunotherapy: 1. GCS together with steroid-sparing agents, IVIG, PE/IA, 2. rituximab, cyclophosphamide; Tumor-therapy: (operation, radiation, chemotherapy)	Chronic disease course: about 50 % may improve or stabilize, about 50 % deteriorate following combined tumor- and immunotherapy (outcome is better in pure anti-Ma2-encephalitis (men) than in combined anti-Ma1/Ma2-encephalitis (women))	[16, 17, 37, 39, 67, 79, 82, 83, 85, 98]
Anti-CV2 (CRMP-3) encephalitis	Age 50–75 years (median 60 years), gender female 60 %, ANPR 30	Lymphocytic pleocytosis (8–70/μl), normal glucose and lactate, mildly elevated protein (47–400 mg/dL), intrathecal IgG synthesis and OCB	Inflammatory infiltrates (perivascular CD4^+^ T cells and B cells, parenchymal CD8^+^ T cells), gliosis, microglia activation, neuronal loss and neuronophagia	Neuronal cell death mediated by neuronal-antigen specific cytotoxic CD8^+^ T cells	Immunotherapy: 1. GCS together with steroid-sparing agents, IVIG, PE/IA, 2. rituximab, cyclophosphamide; Tumor-therapy: (operation, radiation, chemotherapy)	Chronic disease course: usually poor response to immunotherapy and tumor-therapy	[2, 41–43, 67, 79, 83]
Anti-CRMP-5 encephalitis	Age 50–75 years (median 60 years), gender female 60 %, ANPR 150	Lymphocytic pleocytosis (8–370/μl), normal glucose and lactate, mildly elevated protein (47–400 mg/dL), intrathecal IgG synthesis and OCB	Inflammatory infiltrates (perivascular CD4^+^ T cells and B cells, parenchymal CD8^+^ T cells), gliosis, microglia activation, neuronal loss and neuronophagia	Neuronal cell death mediated by neuronal-antigen specific cytotoxic CD8^+^ T cells	Immunotherapy: 1. GCS together with steroid-sparing agents, IVIG, PE/IA, 2. rituximab, cyclophosphamide; Tumor-therapy: (operation, radiation, chemotherapy)	Chronic disease course: usually poor response to immunotherapy and tumor-therapy	[14, 67, 79, 83, 99]
Anti-PCA-1 (Yo) encephalitis	Age 60–70 years (median 65 years), gender female 90 %, ANPR 150	Lymphocytic pleocytosis (median 4/μl, range 1–22/μl),normal glucose and lactate, mildly elevated protein (median 54 mg/dL, range 36–88 mg/dL), intrathecal IgG synthesis and OCB	Inflammatory infiltrates (perivascular CD4^+^ T cells and B cells, parenchymal CD8^+^ T cells and macrophages), gliosis, microglia activation, neuronal loss and neuronophagia	Neuronal cell death mediated by neuronal-antigen specific cytotoxic CD8^+^ T cells	Immunotherapy: 1. GCS together with steroid-sparing agents, IVIG, PE/IA, 2. rituximab, cyclophosphamide; Tumor-therapy: (operation, radiation, chemotherapy)	Chronic disease course: usually poor response to immunotherapy and tumor-therapy	[28, 67, 68, 79, 81, 83, 85]
Anti-PCA-2 encephalitis	Age 45–85 years (median 60 years), gender female 70 %, ANPR 10	Lymphocytic pleocytosis normal glucose and lactate, mildly elevated protein, intrathecal IgG synthesis and OCB	–	Neuronal cell death mediated by neuronal-antigen specific cytotoxic CD8^+^ T cells	Immunotherapy: 1. GCS together with steroid-sparing agents, IVIG, PE/IA, 2. rituximab, cyclophosphamide; Tumor-therapy: (operation, radiation, chemotherapy)	Chronic disease course: usually poor response to immunotherapy and tumor-therapy	[67, 79, 83, 85, 94]
Anti-PCA-Tr encephalitis	Age 15–70 years (median 60 years), gender male 75 %, ANPR 120	Lymphocytic pleocytosis (median 7/μl, range 3–10/μl) normal glucose and lactate, mildly elevated protein (median 41 mg/dL, range 25–72 mg/dL), usually no intrathecal IgG synthesis or OCB	–	Presumably neuronal cell death mediated by neuronal-antigen specific cytotoxic CD8^+^ T cells	Immunotherapy: 1. GCS together with steroid-sparing agents, IVIG, PE/IA, 2. rituximab, cyclophosphamide; Tumor-therapy: (operation, radiation, chemotherapy)	Usually chronic disease course with poor response to immunotherapy and tumor-therapy, a subset of patients may stabilize or improve with eradication of the PCA-Tr antibody following successful tumor-therapy	[5, 8, 9, 22, 37, 67, 79, 83, 85]
Anti-amphiphysin encephalitis	Age 50–80 years (median 65 years), gender female 60 %, ANPR 100	Lymphocytic pleocytosis (median 22/μl, range 2–42/μl), normal glucose and lactate, mildly elevated protein (median 104 mg/dL, range 57–151 mg/dL), intrathecal IgG synthesis and OCB	Inflammatory infiltrates (perivascular CD4^+^ T cells and B cells, parenchymal CD8^+^ T cells and macrophages), gliosis, microglia activation, neuronal loss and neuronophagia	Neuronal cell death and functional impairment mediated by neuronal-antigen specific cytotoxic CD8^*+*^ T cells alternatively: Binding to the amphiphysin protein and internalization of the IgG antibody into the presynaptic ending with disturbance of GABAergic > glutamatergic synaptic transmission	Immunotherapy: 1. GCS together with steroid-sparing agents, IVIG, PE/IA, 2. rituximab, cyclophosphamide; Tumor-therapy: (operation, radiation, chemotherapy)	Chronic disease course: usually poor response to immunotherapy and tumor-therapy	[21, 26, 30, 67, 76, 79, 83, 91]
Anti-GAD 65 encephalitis	Age 15–80 years (median 60 years), gender female 80 %, ANPR 200	Lymphocytic pleocytosis, normal glucose and lactate, mildly elevated protein intrathecal IgG synthesis and OCB	Inflammatory infiltrates (perivascular CD4^+^ T cells and B cells, parenchymal CD8^+^ T cells and macrophages), gliosis, microglia activation, neuronal loss and neuronophagia	Neuronal cell death and functional impairment mediated by neuronal-antigen specific cytotoxic CD8^+^ T cells	Immunotherapy: 1. GCS together with steroid-sparing agents, IVIG, PE/IA, 2. rituximab, cyclophosphamide; Tumor-therapy: occasionally	Chronic disease course: usually poor response to immunotherapy (and tumor-therapy)	[60, 67, 77, 79, 83, 84, 89, 90]
Anti-GABA_A_-receptor-associated protein encephalitis	–,	–	–	Neuronal cell death and functional impairment mediated by neuronal-antigen specific cytotoxic CD8^+^ T cells alternatively: binding to the GABARAP and internalization of the IgG antibody into the postsynaptic density, impairment of GABA_A_-receptor clustering and disturbance of GABAergic synaptic transmission	Immunotherapy: 1. GCS together with steroid-sparing agents, IVIG, PE/IA, 2. rituximab, cyclo-phosphamide	–	[80]
	ANPR 20						
Anti-gephyrin encephalitis	–,	Normal	–	Neuronal cell death and functional impairment mediated by neuronal-antigen specific cytotoxic CD8^+^ T cells alternatively: binding to the gephyrin protein and internalization of the IgG antibody into the postsynaptic density, impariment of GABA_A_- and glycine-receptor clustering and disturbance of GABAergic and glycinergic synaptic transmission	Immunotherapy: 1. GCS together with steroid-sparing agents, IVIG, PE/IA, 2. rituximab, cyclo-phosphamide; Tumor-therapy: (operation, radiation, chemotherapy)	Complete remission upon tumor removal	[10]
	ANPR : 1						

However, there exists a group of CNS disorders with antibodies against intracellular neuronal antigens located mainly at presynaptic (GAD65) or postsynaptic (GABARAP, Gephyrin) sites of inhibitory GABAergic and glycinergic synapses, which less frequently associate with tumors. In these entities, there is often no evidence for cellular or humoral neuronal cytotoxicity, although some patients show neuroaxonal swelling, chromatolysis and vacuolization of neurons, microglial proliferation, as well as infiltration and apposition of cytotoxic CD8^+^ T cells to neurons [6, 40]. Further, there are reports of potentially pathogenic humoral mechanisms probably targeting inhibitory CNS neuronal networks in anti-GAD encephalitis [29, 57, 61], but until now the specificity of possible pathogenic antibodies has not been elucidated. These findings together with the wide spectrum of clinical presentations suggest that anti-GAD encephalitis comprises of a quite heterogenous group of CNS disorders with regard to their etiologies and disease mechanisms.

## Paraneoplastic and non-paraneoplastic autoimmune encephalitis is probably mediated by antibodies to neuronal surface membrane antigens

Autoimmune inflammatory CNS disorders associated with IgG antibodies in the serum and CSF directed against neuronal surface membrane antigens [54, 97] occur both in a paraneoplastic and non-paraneoplastic context. Tumors assumed to drive the pathogenic immune response usually contain neuronally differentiated tissue expressing the respective neuronal antigen or occur in organs with a role in immune regulation, such as the thymus.

Antibodies bind to synaptic and extra-synaptic ligand- and voltage-gated ion channels (Table 2) involved in excitatory (AMPA-, NMDA-, mGluR1-, mGluR5-, and nAch-receptors, VGCC, VGKC) and inhibitory (GABA_B_- and Glycine-receptors, VGKC) synaptic transmission and plasticity. Moreover, these antibodies also target neuronal membrane proteins implicated in clustering of voltage-gated potassium channels inside the synapse [leucine-rich glioma-inactivated 1 (LGI1)] or outside the synapse at the juxtaparanodal region of the node of Ranvier [contactin-2 and contactin-associated protein-like 2 (CASPR2)] thereby indirectly impacting neuronal excitability.

**Table 2:** Encephalitis associated with antibodies against neuronal surface membrane antigens (modified and extended from Melzer et al. [70])

Entity	Patients	Triggers	Clinical hallmarks	Imaging	Electrophysiology	Laboratory	
Anti-NMDA-R encephalitis	Age 1–80 years (median 20 years), gender female 80, ANPR 500	Tumors (age-, gender-, race-dependent, about 50 %): ovary/testis (teratoma), breast, lung (SCLC), lymphoma	Multistage cortico-subcortical encephalopathy: 1. Prodromal phase (days) often with (viral) infections, 2. Psychiatric symptoms (1–2 weeks): psychosis, confusion, amnesia, dysphasia, 3. neurological symptoms (weeks): movement disorders (choreoathetoid, mute, catatonic), autonomic instability, respiratory failure, reduced consciousness, seizures, 4. recovery of symptoms in reverse of their appearance	MRI: no correlating signal abnormalities (50 %), transient T2/FLAIR hyperintense signal in cerebral cortex cerebellar cortex, basal ganglia, brainstem, spinal cord, Gd-enhancement in cortical meninges, basal ganglia, frontotemporal or mediotemporal cortical atrophy (50 %)	EEG: 1. focal or widespread interictal and ictal epileptiform activity, 2. generalized slowing	Anti-NMDA-R (NR1/NR2) IgG1/3-antibody in serum and CSF, intrathecal anti-NMDA-R IgG1/3 synthesis, titers correlate well with clinical disease course/therapy tumors often express NMDA-R	
Anti-AMPA-R encephalitis	Age 40–80 years (median 60 years), gender female 90 %, ANPR 15	Tumors (about 70 %); thymus (thymoma), breast, lung (SCLC, non-SCLC)	Limbic encephalitis: 1. focal temporal lobe and secondary generalized seizures, 2. short-term memory loss/disorientation, 3. psychiatric symptoms (psychosis) evolving within days–weeks	MRI: T2/FLAIR hyperintense signal in one or both medial temporal lobes (often asymmetric), rarely Gd-enhancement (90 %)	EEG: 1. focal interictal and ictal epileptiform activity in one or both temporal lobes, 2. focal or generalized slowing	Anti-AMPA-R (GluR1/2) IgG antibody in serum and CSF, intrathecal anti-AMPA-R IgG synthesis, titers correlate with clinical disease course/therapy tumors often express AMPA-R	
Anti-GABA_B_-R encephalitis	Age 25–75 years (median 60 years), gender female 50 %, ANPR 25	Tumors (about 60 %): lung (SCLC, non-SCLC), thymus (thymoma)	Limbic encephalitis with prominent seizures: 1. focal temporal lobe and secondary generalized seizures, 2. short-term memory loss/disorientation, 3. psychiatric symptoms (psychosis) evolving within days–weeks	MRI: T2/FLAIR hyperintense signal in one or both medial temporal lobes (often asymmetric), rarely Gd-enhancement (70 %)	EEG: 1. focal interictal and ictal epileptiform activity in one or both temporal lobes, 2. focal or generalized slowing	Anti-GABA_B_-R (GABA_B1_) IgG1 antibody in serum and CSF, intrathecal anti-GABA_B_-R IgG1 synthesis, correlation of titers with clinical disease course/therapy not yet determined expression of GABA_B_-R by tumors not yet determined	
Anti-Glycine-R encephalitis	Age 30–60 years (median 50 years), gender male 80 %, ANPR 4	Tumors: typically none (thymoma)	Hyperekplexia, stiff-person syndrome, progressive encephalomyelitis with rigidity and myoclonus (PERM): stiffness/rigidity, excessive startle, brainstem dysfunction	MRI: typically normal	EMG: excessive startle response, continuous involuntary motor activity	Anti-Gly-R (GlyRα1) IgG1 antibody in serum and CSF, intrathecal anti-Gly-R IgG1 synthesis, titers seem to correlate with clinical disease course/therapy	
Anti-VGKC complex encephalitis: LGI1	Age 30–80 years (median 60 years), gender male 65 %, ANPR 120	Tumors: (about 10 %): thymus (thymoma), lung (SCLC)	Limbic encephalitis: 1. focal temporal lobe and secondary generalized seizures, 2. short-term memory loss/disorientation, 3. psychiatric symptoms (psychosis) evolving within days–weeks	MRI: T2/FLAIR hyperintense signal in one or both medial temporal lobes (often asymmetric), rarely Gd-enhancement	EEG: 1. interictal focal epileptiform activity or slowing over one or both temporal lobes, 2. ictal focal or generalized epileptiform activity	Anti-LGI1 IgG4/1 antibody in serum and CSF, intrathecal anti-LGI1 IgG4/1 synthesis infrequent, correlation of titers with clinical disease course/therapy not yet determined expression of LGI1 by tumors not yet determined SIADH with hyponatremia (115–130 mmol/l)	
Anti-VGKC complex encephalitis: CASPR2	Age 45–80 years (median 60 years), gender male 85 %, ANPR 40	Tumors (about 10 %): thymus (thymoma), lung (SCLC)	Morvan’s syndrome: 1. psychiatric disturbance 2. seizures, 3. sleep disturbance (insomnia), 4. dysautonomia, 5. neuromyotonia in various combinations cerebellitis	MRI: T2/FLAIR hyperintense signal in one or both medial temporal lobes (often asymmetric), rarely Gd-enhancement (about 40 %)	EEG: 1. focal or generalized interictal and ictal epileptiform activity, 2. focal or generalized slowing EMG: spontaneous doublet, triplet or multiplet single-unit discharges	Anti-CASPR2 IgG4/1 antibody in serum and CSF, intrathecal anti-CASPR2 IgG4/1 synthesis not determined, correlation of titers with clinical disease course/therapy not yet determined expression of CASPR2 by tumors not yet determined SIADH with hyponatremia (115–130 mmol/l)	
Anti-mGlu-R1 encephalitis	Age 20–50 years, gender female 100 %, ANPR 3	Tumors: none or Hodgkin lymphoma (in remission)	Cerebellitis	MRI: normal or T2/FLAIR hyperintense signal and atrophy of the cerebellum	–	Anti-mGlu-R1 IgG antibody in serum and CSF, intrathecal anti-mGlu-R1 IgG synthesis, correlation of titers with clinical disease course/therapy not yet determined	
Anti-mGlu-R5 encephalitis	Age 15–45 years, gender female 50 %, ANPR 2	Tumors: Hodgkin lymphoma	Limbic encephalitis (Ophelia syndrome): 1. focal temporal lobe and secondary generalized seizures, 2. short-term memory loss/disorientation, 3. psychiatric symptoms (psychosis) evolving within days–weeks	MRI: normal or T2/FLAIR hyperintense signal in one or both medial temporal lobes and other cortical and subcortical gray matter areas	EEG: 1. interictal focal epileptiform activity or slowing over one or both temporal lobes, 2. ictal focal or generalized epileptiform activity	Anti-mGlu-R5 IgG antibody in serum and CSF, intrathecal anti-mGlu-R5 IgG synthesis not determined, correlation of titers with clinical disease course/therapy not yet determined	
Anti-P/Q type/N-type VGCC encephalitis	Age 30–80 years, gender male 80 %, ANPR 120	Tumors: (about 50 %) lung (SCLC), breast, ovary	Cerebellitis, Lambert–Eaton myasthenic syndrome (LEMS)	MRI: normal or T2/FLAIR hyperintense signal and atrophy of the cerebellum	EMG: decrement of compound muscle action potential on 2–5/s repetitive nerve stimulation, increment of compound muscle action potential on 30–50/s repetitive nerve stimulation or “post-tetanic” stimulation	Anti-P/Q type/N-type VGCC IgG antibody in serum and CSF, intrathecal anti-P/Q type/N-type VGCC IgG synthesis, correlation of titers with clinical disease course/therapy not yet determined expression of VGCC by tumors not yet determined	
Anti-nAch-R encephalitis	Age 17–103 years (median 65 years), gender male (55 %), ANRP 150	Tumors (carcinoma in about 30 %): lung (non-SCLC, SCLC), breast, ovary, uterus, prostate, colon, thyroid, kidney, bladder, thymus (thymoma), melanoma	Cortical encephalitis, basal ganglionitis, dysautonomia peripheral (sensory, motor, sensorimotor, autonomic) neuropathy	MRI: usually normal, occasionally T2/FLAIR hyperintense signal in basal ganglia	EEG: generalized slowing Nerve conduction studies: predominantly axonal sensory, motor or sensorimotor neuropathy	Anti-nAch-R IgG antibody in serum and CSF, intrathecal anti- nAch-R IgG synthesis not yet determined, strong correlation of titers with clinical disease course/therapy expression of nAch-R by tumors not yet determined	

Entity	Patients	CSF	Neuropathology	Putative disease mechanisms	Therapy	Disease course and prognosis	References
Anti-NMDA-R encephalitis	Age 1–80 years (median 20 years), gender female 80, ANPR 500	Lymphocytic pleocytosis (median 32/μl, range 5–480/μl), normal glucose and lactate, mildly elevated protein (median 67 mg/dl, range 49–213 mg/dl), intrathecal IgG synthesis and OCB	Microglia activation, perivascular B cell/plasma cell infiltrates, rare T cell infiltrates	Reversible IgG1/3-antibody-mediated NMDA-R crosslinking and internalization, alteration in glutamatergic synaptic transmission and plasticity, no neuronal cell death	Immunotherapy: 1. GCS together with steroid-sparing agents, IVIG, PE/IA, 2. rituximab, cyclophosphamide; tumor therapy: (operation, radiation, chemotherapy)	Monophasic or relapsing-remitting disease course: prolonged recovery over weeks–months (incomplete, spontaneous remission, accelerated and more complete remission under immunotherapy/tumor removal), frequent relapses (about 25 %) in patients without tumor or with insufficient immunotherapy	[15, 18, 20, 44, 47, 72, 100]
Anti-AMPA-R encephalitis	Age 40–80 years (median 60 years), gender female 90 %, ANPR 15	Lymphocytic pleocytosis (median 24/μl; range, 6–75/μl), normal glucose and lactate, normal–mildly elevated protein (median 51 mg/dl, range <46–420 mg/dl), intrathecal IgG synthesis and OCB	–	Reversible IgG-antibody-mediated AMPA-R crosslinking and internalization, alteration in glutamatergic synaptic transmission and plasticity, no neuronal cell death	Immunotherapy: 1. GCS together with steroid-sparing agents, IVIG, PE/IA, 2. rituximab, cyclophosphamide; Tumor therapy: (operation, radiation, chemotherapy)	Monophasic or relapsing-remitting disease course: good response to immunotherapy/tumor removal, frequent relapses (about 50 %) in patients without tumor or with insufficient immunotherapy	[32, 50, 72]
Anti-GABA_B_-R encephalitis	Age 25–75 years (median 60 years), gender female 50 %, ANPR 25	Lymphocytic pleocytosis (median 20/μl; range, 0–950/μl), normal glucose and lactate, normal–mildly elevated protein (median 35 mg/dl, range 22–109 mg/dl), intrathecal IgG synthesis and OCB	–	IgG1-antibody-mediated GABA_B_-R blockade without internalization, alteration in GABAergic synaptic transmission and plasticity, impairment of pre- and postsynaptic GABAergic inhibition, no neuronal cell death	Immunotherapy: 1. GCS together with steroid-sparing agents, IVIG, PE/IA, 2. rituximab, cyclophosphamide; Tumor therapy: (operation, radiation, chemotherapy)	Monophasic or chronic disease course: good response to immunotherapy/tumor removal, rare relapses	[7, 53, 72]
Anti-Glycine-R encephalitis	Age 30–60 years (median 50 years), gender male 80 %, ANPR 4	CSF normal or lymphocytic pleocytosis (median 33/μl, range 0–60/μl), normal glucose and lactate, normal protein, intrathecal IgG synthesis/OCB	–	IgG1-antibody-mediated Gly-R blockade, alteration in glycinergic synaptic transmission and plasticity	Immunotherapy: 1. GCS together with AZT/MMF, IVIG, PE/IA, 2. rituximab, cyclophosphamide, (tumor therapy)	Monophasic or relapsing disease course: good response to immunotherapy, occasional relapses	[45, 64, 72]
Anti-VGKC complex encephalitis: LGI1	Age 30–80 years (median 60 years), gender male 65 %, ANPR 120	CSF normal or lymphocytic pleocytosis, normal glucose and lactate, normal or mildly elevated protein (median 35 mg/dl range up to 44 mg/dl), intrathecal IgG synthesis/OCB	–	IgG4/1-antibody mediated disruption of the presynaptic VGKC complex and altered synaptic transmission	Immunotherapy: 1. GCS together with AZT/MMF, IVIG, PE/IA, 2. rituximab, cyclophosphamide, (tumor therapy)	Monophasic or relapsing disease course: good response to immunotherapy, occasional relapses	[46, 51, 72]
Anti-VGKC complex encephalitis: CASPR2	Age 45–80 years (median 60 years), gender male 85 %, ANPR 40	CSF normal or lymphocytic pleocytosis (median 3/μl, range 0–15/μl), normal glucose and lactate, normal or mildly elevated protein (median 35 mg/dl range up to 44 mg/dl), intrathecal IgG synthesis/OCB	–	IgG4/1-antibody mediated disruption of the para/juxtanodal VGKC complex and altered neuronal excitability	Immunotherapy: 1. GCS together with AZT/MMF, IVIG, PE/IA, 2. rituximab, cyclophosphamide; (tumor therapy)	Monophasic or relapsing disease course: spontaneous remission, good response to immunotherapy, relapses may occur	[4, 46, 52, 72]
Anti-mGlu-R1 encephalitis	Age 20–50 years, gender female 100 %, ANPR 3	CSF normal or lymphocytic pleocytosis (range 28–190/μl), normal glucose and lactate, normal or mildly elevated protein (range 28–72 mg/dl), intrathecal IgG synthesis/OCB	Purkinje cell loss with amputation of the dendritic tree, astrogliosis, no inflammatory cell infiltrates	IgG-antibody binding to mGlu-R1 at the perisynaptic site of Purkinje cell dendritic spines, impairment of cerebellar synaptic plasticity and motor learning, Purkinje cell death	Immunotherapy: 1. GCS together with AZT/MMF, IVIG, PE/IA, 2. rituximab, cyclophosphamide;	Monophasic or chronic disease course: good response to immunotherapy	[13, 62, 88]
Anti-mGlu-R5 encephalitis	Age 15–45 years, gender female 50 %, ANPR 2	Lymphocytic pleocytosis (range 23–114/μl), normal glucose and lactate, normal or mildly elevated, protein (range 40–55 mg/dl), intrathecal IgG synthesis/OCB	–	IgG-antibody binding to mGlu-R5 on hippocampal neurons, impairment of synaptic plasticity, learning and memory	Immunotherapy: GCS together with AZT/MMF, IVIG, PE/IA Tumor therapy	Monophasic or chronic disease course: good response to immunotherapy/tumor therapy	[11, 55]
Anti-P/Q type/N-type VGCC encephalitis	Age 30–80 years, gender male 80 %, ANPR 120	CSF normal or lymphocytic pleocytosis, normal glucose and lactate, normal or mildly elevated protein, intrathecal IgG synthesis/OCB	Purkinje cell loss, cerebellar cortical gliosis, rare or no inflammatory infiltrates in the cerebellum	IgG-antibody binding to P/Q type/N-type VGCC on central and peripheral neurons, impairment of neurotransmitter release, neuronal cell death	Immunotherapy: 1. GCS together with steroid-sparing agents, IVIG, PE/IA, 2. rituximab, cyclophosphamide; tumor therapy: (operation, radiation, chemotherapy)	Chronic disease course: limited response to immuno-therapy/tumor therapy	[27, 34, 56, 65]
Anti-nAch-R encephalitis	Age 17–103 years (median 65 years), gender male (55 %), ANRP 150	CSF normal or lymphocytic pleocytosis (range 10–100/μl), normal glucose and lactate, normal or mildly elevated protein (median, 63 mg/dl; range, 48–181 mg/dl), intrathecal IgG synthesis/OCB	–	IgG-antibody binding to nAch-R on central and peripheral neurons, impairment of synaptic transmission	Immunotherapy: 1. GCS together with steroid-sparing agents, IVIG, PE/IA, 2. rituximab, cyclophosphamide, tumor therapy: (operation, radiation, chemotherapy)	Monophasic or chronic disease course: good response to immuno-therapy/tumor therapy	[3, 31, 66, 95]

*AMPA* 2-amino-3-(5-methyl-3-oxo-1,2-oxazol-4-yl)propanoic acid, *ANPR* approximate number of patients reported, *AZA* azathioprine, *CSF* cerebrospinal fluid, *EEG* electroencephalography, *EMG* electromyography, *FLAIR* fluid attenuated inversion recovery, *GABA* γ-aminobutyric acid, *GABARAP* γ-aminobutyric acid receptor associated protein, *GCS* glucocorticosteroids, *IA* immunoadsorption, *IVIG* intravenous immunoglobulins, *mGluR* metabotropic glutamate receptor, *MMF* mycophenolate mofetil, *n-Ach* nicotinic acetylcholine, *NMDA* N-methyl-D-aspartate, *OCB* oligoclonal bands, *PE* plasma exchange, *SIADH* syndrome of inappropriate antidiuretic hormone secretion, *SCLC* small cell lung cancer, *VGCC* voltage-gated calcium channels, *VGKC* voltage-gated potassium channels

In principle, depending on the IgG subtype, antibodies may (1) specifically activate or block the function of their target molecules (GABA_B_-, Glycine- nAch-receptors, VGCC, VGKC), (2) crosslink and internalize the receptors (AMPA- and NMDA-receptors), (3) activate the complement cascade with subsequent formation of the terminal membrane attack complex and target cell lysis (probably mGluR1/5 receptors, VGCC, VGKC) and (4) activate Fc-receptors with subsequent antibody-dependent cell-mediated cytotoxicity (ADCC) mainly by NK cells [23]. However, the effector mechanisms involved in the pathogenic effect of the autoantibodies within the CNS are not yet fully understood. In fact, autoantibodies in anti-NMDA-R and -AMPA-R, GABA_B_-R encephalitis are of the IgG1 and IgG3 type and are thus capable of activating complement in the presence of patient plasma (containing high concentrations of complement factors). However, in none of the reported autopsy or biopsy studies complement depositions could be detected on neurons suggesting that in the presence of patient CSF (containing low concentrations of complement factors) these IgG 1 and 3 autoantibodies do not lead to relevant complement activation [6, 32, 47, 50, 63, 93]. In contrast, autoantibodies in VGKC-complex encephalitis are predominantly of the IgG4 (and IgG1) type and thus are unable to activate complement in the presence of patient plasma. However, in the only biopsy study reported thus far, complement depositions could be detected on neurons in VGKC-complex encephalitis suggesting that in the presence of patient CSF these IgG4 (or the IgG1) autoantibodies are capable of activating complement [6]. These conflicting results suggest that effector mechanisms of distinct autoantibody classes and subtypes may differ between the CNS and peripheral organs.

Moreover, with the growing clinical awareness of the autoantibodies and improved diagnostic possibilities due to cell-based detection systems more patients are currently identified with persisting IgM antibodies without subsequent class switch to IgG and rarely with IgA antibodies against neuronal plasma membrane antigens [78]. This class-diversity of the neuron-directed adaptive humoral immune response in CNS may indeed reflect different milieus (e.g., absence or presence of different tumors), in which B cell activation takes place. Moreover, lack of class switch might indicate insufficient CD4^+^ T cell-mediated help in B cell activation. However, these issues need further experimental investigation. Further, it remains to be determined, whether cytotoxicity by CD8^+^ T cells contributes to functional and structural neuronal impairment.

Inflammatory CNS disorders associated with IgG antibodies against neuronal surface membrane antigens are characterized by a presentation of CNS- and sometimes PNS-related clinical symptoms representing the expression distribution and function of the respective target antigen [54, 97]. On cerebral MRI cortical and subcortical gray matter regions may display mild and often transient T2/FLAIR hyperintense signals. CSF findings may initially be normal, but often include inflammatory changes (lymphocytic pleocytosis, mildly elevated protein, intrathecal IgG synthesis with oligoclonal bands, but normal glucose and lactate levels). The clinical course is usually either monophasic or relapsing-remitting, but rarely progressive and clinical symptoms and paraclinical measures usually display a good response to immunotherapy, especially to antibody-depleting therapies. Upon detection of a tumor, sufficient tumor therapy is crucial to halt the pathogenic immune response [54, 97].

It should be noted that antibody-mediated autoimmunity to non-neuronal ion channels on the cell surface membrane [aquaporin 4 [74] on astrocytes (and neurons), Na(x) channel [38] on ependymal cells, astrocytes, and pituicytes (circumventricular organs)] may occur also in a paraneoplastic context (neuromyelitis optica: breast cancer, Hürthle cell thyroid carcinoma, carcinoid, pituitary somatotropinoma, B cell lymphoma, monoclonal gammopathy [74]; essential hypernatremia [38]: ganglioneuroma).

## Conclusions

A growing number of immune-mediated CNS disorders of paraneoplastic and non-paraneoplastic autoimmune origin have recently emerged, in which neurons are the target of both adaptive cellular and humoral immune responses. In autoimmune encephalitis associated with antibodies to neuronal surface membrane antigens, potentially reversible mechanisms of antibody-mediated impairment of synaptic transmission and neuronal excitability prevail. Hence, these disorders offer unique insight and provoke further investigation into the consequences of immune-mediated disruption of distinct neuronal signaling pathways within the living CNS.

In contrast, paraneoplastic autoimmune encephalitis associated with antibodies to intracellular neuronal antigens seems to be mediated by cytotoxic CD8^+^ T cells that cause functional and structural neuronal impairment in a way not specific for the respective antigen.
